# QTL-seq for identification of loci associated with resistance to *Phytophthora* crown rot in squash

**DOI:** 10.1038/s41598-020-62228-z

**Published:** 2020-03-24

**Authors:** Alexis Ramos, Yuqing Fu, Vincent Michael, Geoffrey Meru

**Affiliations:** Horticultural Sciences Department and Tropical Research and Education Center, University of Florida, 18905 SW 280th St, Homestead, FL 33031 USA

**Keywords:** Agricultural genetics, Plant breeding

## Abstract

*Phytophthora capsici* Leonian, the causal agent of foliar blight, root rot, fruit rot and crown rot syndromes in squash (*Cucurbita moschata*), is a devastating pathogen worldwide. Resistance to *Phytophthora* crown rot in University of Florida breeding line #394-1-27-12 (*C. moschata*) is conferred by three independent dominant genes (R1R2R3). Availability of DNA markers linked to R1R2R3 genes would allow efficient breeding for *Phytophthora* crown rot resistance through marker-assisted selection (MAS). The goal of the current study was to identify quantitative trait loci (QTLs) associated with resistance to *Phytophthora* crown rot in an F_2_ population (n = 168) derived from a cross between #394-1-27-12 (R) and Butter Bush (S) using QTL-seq bulk segregant analysis. Whole-genome resequencing of the resistant (n = 20) and susceptible (n = 20) bulk segregants revealed **~**900,000 single nucleotide polymorphisms distributed across *C. moschata* genome. Three QTLs significantly (P < 0.05) associated with resistance to *Phytophthora* crown rot were detected on chromosome 4 (*QtlPC-C04*), 11 (*QtlPC-C11*) and 14 (*QtlPC-C14*). Several markers linked to these QTLs are potential targets for MAS against *Phytophthora* crown rot in *C. moschata*. The present study reports the first QTLs associated with *Phytophthora* crown rot resistance in *C. moschata*.

## Introduction

Disease epidemics caused by the oomycete *Phytophthora capsici* Leonian are a major challenge for squash (*Cucurbita pepo* L*., C. moschata* Duchesne, and *C. maxima* Duchesne) growers worldwide^1^. The pathogen causes foliar blight, root rot, fruit rot and crown rot syndromes, and is particularly severe under flooding conditions, often resulting in total crop loss^2^. Current strategies for managing *P. capsici* in commercial squash production rely heavily on chemical fungicides, however, existence of fungicide-resistant *P. capsici* isolates in major squash growing regions has rendered many chemicals ineffective for the control of the pathogen^3–5^. Cultural management practices such as crop rotation and soil-water management focus on inoculum reduction or avoidance, but are not solely effective, particularly under heavy disease pressure^6^. Host resistance is the best strategy for managing this disease, but no commercial cultivars resistant to the pathogen are currently available^7^ to support the U.S. squash industry currently valued at 230 million dollars annually^8^.

Extensive efforts have led to identification of sources of resistance to *Phytophthora* crown rot in unimproved germplasm of *Cucurbita*. Padley *et al*.^9^ identified sixteen plant introductions (PIs) of *C. pepo* that showed moderate to high resistance to *Phytophthora* crown rot. Among these, PIs 181761 and 615132 were the most resistant (disease severity (DS) ≤ 1.3 out of 5)^9^. In *C. moschata*, Chavez and Kabelka^10^ identified five PIs (176531, 458740, 442266, 442262 and 634693) that exhibited high resistance (DS ≤ 1 out of 5) to *Phytophthora* crown rot. Kabelka *et al*.^11^ identified a source of resistance in *C. lundeliana* that was successfully introgressed into a *C. moschata* breeding line #394-1-27-12^12^.

An inheritance study using F_2_ and backcross populations revealed that resistance in breeding line #394-1-27-12 is conferred by three independent dominant genes (R1R2R3), all of which must be present to confer resistance against the pathogen^12^. Despite availability of resistance in #394-1-27-12 for more than a decade, it remains unexploited in commercial cultivars. Marker-assisted selection (MAS) for *Phytophthora* crown rot resistance in #394-1-27-12 would greatly expedite development and release of resistant commercial cultivars. However, the genetic loci associated with *Phytophthora* crown rot resistance in #394-1-27-12 are currently unknown.

Bulk segregant analysis (BSA) is a powerful tool for rapid identification of DNA markers linked to a trait of interest^13,14^. The QTL-seq method combines BSA and next generation sequencing (whole-genome resequencing) to identify, fine map, and improve resolution of linked QTL^15^. The QTL-seq approach has been successfully applied to identify loci associated with economically important traits in crops such as rice^15^, cucumber^16,17^, tomato^18^, chickpea^19,20^, peanut^21^, watermelon^22,23^, and broccoli^24,25^.

The goal of the current study was to use QTL-seq to identify QTLs associated with resistance to *Phytophthora* crown rot in an F_2_ population derived from a cross between the resistant breeding line #394-1-27-12 and Butterbush, a susceptible butternut-type cultivar.

## Results

### Phenotypic data

**Figure 1:**
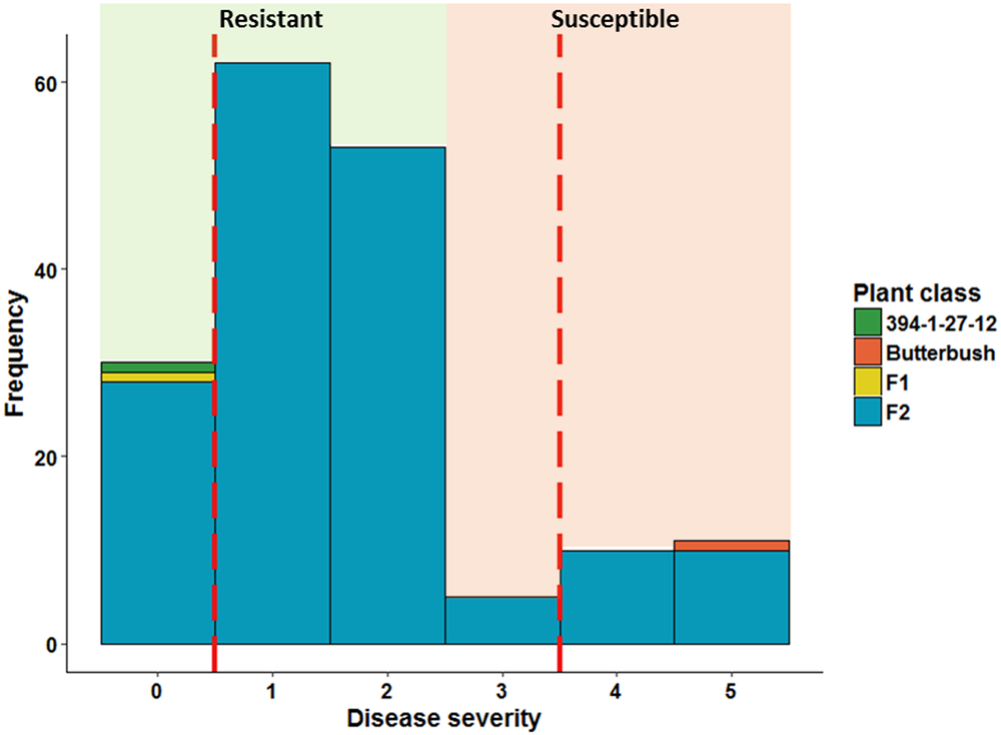
Disease severity in the parents, F_1_, and F_2_ individuals. Red dashed vertical lines indicate cutoff for resistant (disease score of 0) and susceptible (disease score of 4 and 5) individuals used for DNA bulking and sequencing. Green, red, yellow and blue bars represent #394-1-27-12, Butterbush, F_1_ and F_2_ plants, respectively.

Breeding line #394-1-27-12 (mean DS = 0) and the F_1_ (mean DS = 0) individuals exhibited high resistance to *Phytophthora* crown rot (Fig. 1), and grew vigorously throughout the duration of the experiment. In contrast, the susceptible parent (Butterbush; mean DS = 5) rapidly succumbed to the pathogen. As expected, the F_2_ population (mean DS = 1.6 ± 1.3) segregated into susceptible and resistant classes, in varying degree of both. No transgressive segregation was observed in either direction (Fig. 1).

### QTL-seq analysis

High-throughput sequencing of the libraries generated 342.95 to 399.49 million reads per sample, with a read mapping ratio of >98%, irrespective of the consensus reference genome used (Table 1). The coverage ranged from 45.16 to 52.62 per sample (Table 1), while the Q20 exceeded 97% across all samples (data not shown). Each of the bulk sequences was aligned to the consensus reference genomes (consensus fasta files of Butterbush and #394-1-27-12), revealing 987,669 and 901,184 SNPs, respectively. The mean coverage across all samples was 45X.

**Table 1:** Whole genome mapping statistics for the parents and bulks.

Sample	Consensus reference genome^a^	Total reads	Mapped reads	Mapping ratio (%)	Properly paired (%)	Average coverage (x)
R_bulk	#394-1-27-12	399,464,448	394,662,016	98.80	91.28	52.62
S_bulk	#394-1-27-12	342,955,380	338,758,574	98.78	91.30	45.17
R_bulk	Butterbush	399,492,596	394,578,517	98.77	91.25	52.61
S_bulk	Butterbush	342,981,424	338,708,743	98.75	91.29	45.16

^a^The consensus reference genomes were created by substituting alleles in the published squash reference genome *C. moschata* cv. Rifu with the respective parental alleles.

**Figure 2:**
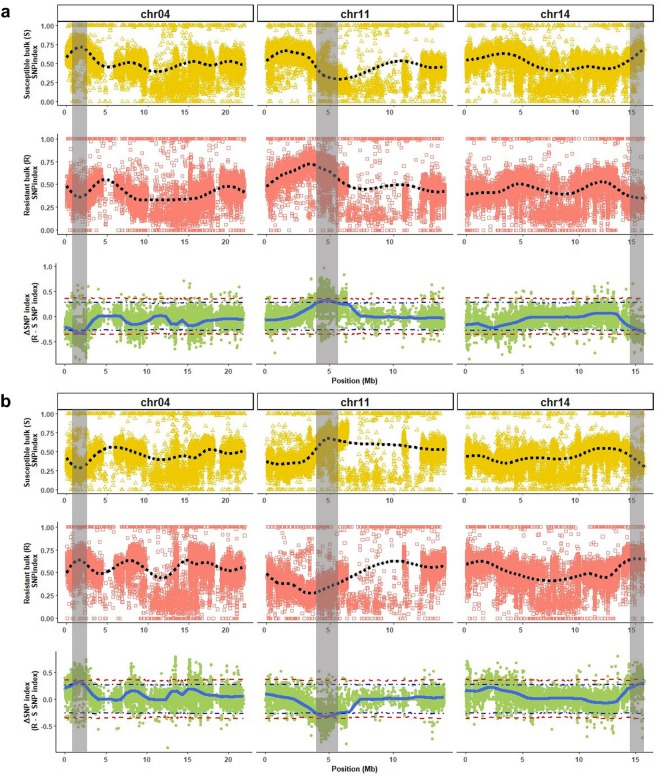
Quantitative trait loci (QTL) (highlighted in gray) associated with *Phytophthora* crown rot resistance in *Cucurbita moschata* on chromosome (chr) 4, 11 and 14 using either #394-1-27-12 (**a**) or Butterbush (**b**) as consensus reference genome. The black dotted lines represent the smoothed conditional mean for Susceptible (S) and Resistant (R) bulks SNP indexes, while the blue line represents the tricubeΔSNP for the ΔSNP index. The purple and red dotted lines in the ΔSNP index plot are the 95% and 99% confidence intervals for the regions, respectively.

QTL-seq analysis detected three QTL on chromosomes 4 (*QtlPC-C04*), 11 (*QtlPC-C11*), and 14 (*QtlPC-C14*) that were significantly (surpassed 95% confidence interval) associated with resistance to *Phytophthora* crown rot in *C. moschata* (Table 2, Fig. 2 and Supplementary Figs. 1 and 2). The three QTLs were detected regardless of the parent used as consensus reference genome; however, there was variation in the significant interval for each region. The interval for the detected QTL was smallest in *QtlPC-C04* (0.58 Mb), and largest in *QtlPC-C11* (1.63 Mb) (Table 2), with an average interval of 1.25 Mb across the three QTL. Irrespective of the parental consensus reference genome used, the position of the highest ΔSNP-index was the same for the QTLs on chromosome 4 and 14, but differed by 6.44 kb for the QTL on chromosome 11 (Table 2). There were 664 genes harbored within the intervals of the three QTL. Among these, 46 were annotated as resistant gene homologs: 24 nucleotide-binding sites leucine-rich repeats, 12 serine/threonine protein kinases and 10-protein phosphatases.

**Table 2:** Quantitative trait loci (*P* < *0.05*) associated with resistance to *Phytophthora* crown rot using either #394-1-27-12 or Butterbush as the consensus reference genome.

Consensus reference genome^a^	Chromosome	Start (bp)	End (bp)	Interval (bp)	Position of most extreme ΔSNP (bp)	Peak ΔSNP index
Butterbush	04	887,645	2,456,537	1,568,892	2,049,406	-0.34
	11	3,992,901	5,600,607	1,607,706	4,813,000	0.32
	14	15,209,401	15,797,562	588,161	15,797,562	-0.30
#394-1-27-12	04	895,380	2,372,777	1,477,397	2,049,406	0.32
	11	3,992,901	5,626,546	1,633,645	4,819,436	−0.33
	14	15,161,862	15,797,562	635,700	15,797,562	0.30

^a^The consensus reference genomes were created by substituting alleles in the published squash reference genome *C. moschata* cv. Rifu with the respective parental alleles.

### Marker test

**Figure 3:**
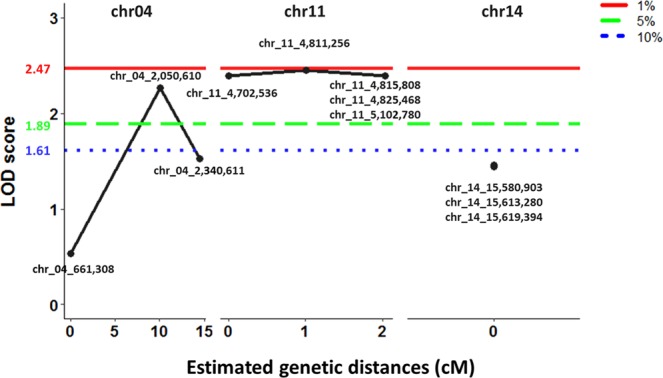
Logarithm of odds (LOD) scores for the genotyped markers in the individuals constituting the susceptible and resistant bulks. The red, green, and blue dotted lines represent the estimated genome wide LOD thresholds (4,000 permutations) for 1%, 5%, and 10% significance levels, respectively. Estimated genetic distance is indicated on the horizontal axis.

**Figure 4:**
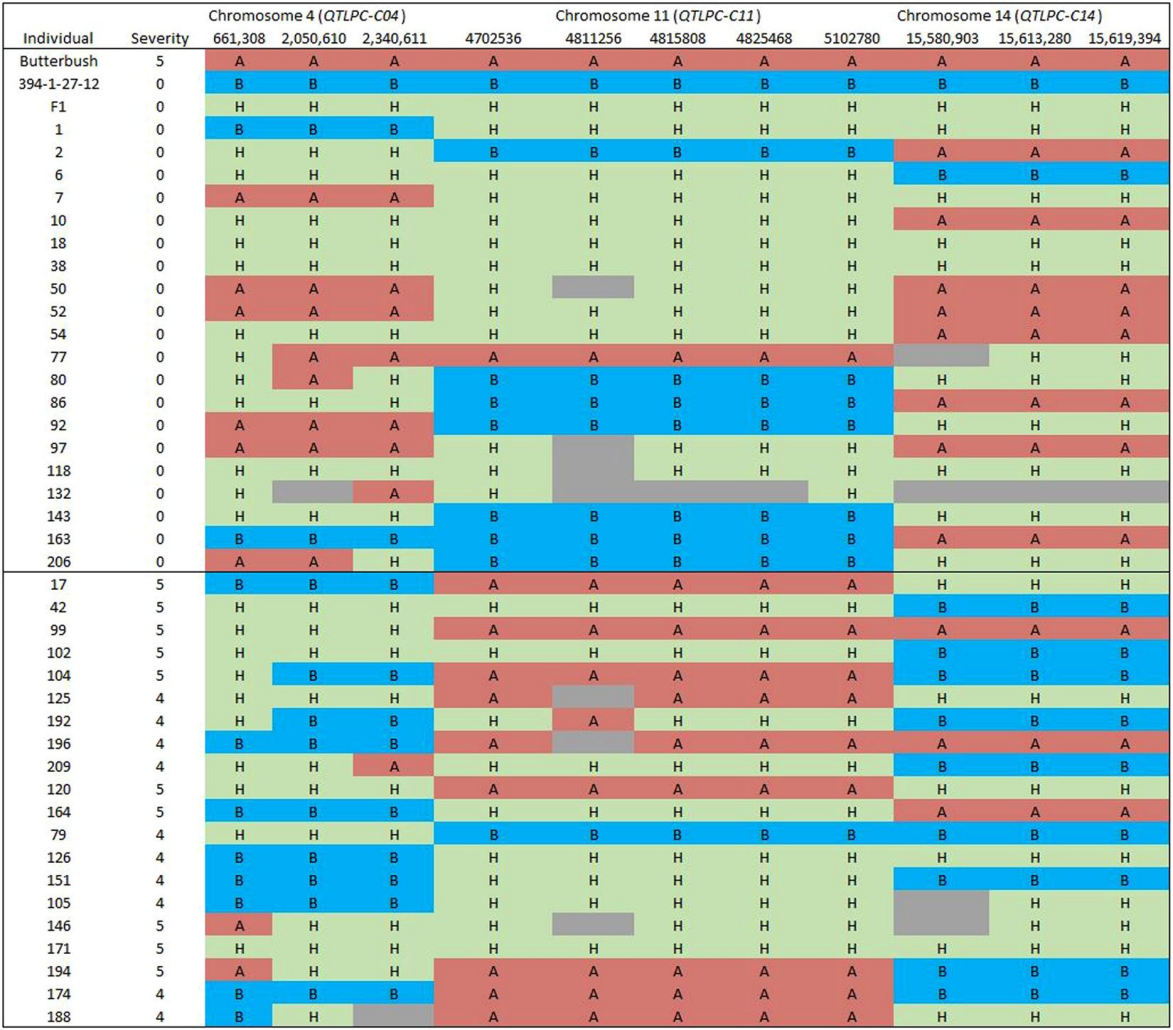
Genotypes across eleven markers for the parents (#394-1-27-12 and Butterbush), F_1_ and F_2_ individuals constituting the susceptible and resistant bulks. A (red shade) and B (blue shade) represent alleles contributed by Butterbush and #394-1-27-12, respectively, while H (green shade) represent heterozygous loci. Missing genotype data is represented by gray shade.

Parents, F_1_ and F_2_ individuals comprising the resistant and susceptible bulks were genotyped with eleven markers. Kruskal-Wallis test indicated that one marker on chromosome 4 (chr_04_2,050,610) and five markers on chromosome 11 (chr_11_4,702,536, chr_11_4,811,256, chr_11_4,815,808, chr_11_4,825,468, chr_11_5,102,780), were significantly associated with resistance to *Phytophthora* crown rot (Table 3). These results were confirmed using non-parametric interval mapping (P < 0.05) (Table 3 and Fig. 3). Although markers adjacent to QtlPC-C14 failed to surpass the significant threshold, their P values were low (P = 0.06-0.07) (Table 3). Multiple QTL mapping revealed no interaction among the three QTLs. Surprisingly, the genotype calls of individuals comprising the resistant and susceptible bulks revealed that both parents contributed alleles for *Phytophthora* crown rot resistance (Fig. 4). #394-1-27-12 (resistant) contributed alleles for resistance from *QtlPC-C11*, while those on *QtlPC-C04* and *QtlPC-C14* were derived from Butterbush (susceptible). Majority of the individuals were heterozygous for the eleven markers targeting the three QTL.

**Table 3:** Chromosomal location and association of markers with *Phytophthora* crown rot resistance in *Cucurbita moschata*.

Marker	Chromosome	Physical position (bp)^a^	Kruskal-Wallis test P value	LOD score	Non-parametric Interval Mapping P value
chr_04_661,308	4	661,308	0.30	0.53	0.787
chr_04_2,050,610	4	2,050,610	0.013*	2.26	0.018*
chr_04_2,340,611	4	2,340,611	0.07	1.53	0.119
chr_11_4,702,536	11	4,702,536	0.004**	2.39	0.013*
chr_11_4,811,256	11	4,811,256	0.011*	2.45	0.012*
chr_11_4,815,808	11	4,815,808	0.008**	2.39	0.013*
chr_11_4,825,468	11	4,825,468	0.008**	2.39	0.013*
chr_11_5,102,780	11	5,102,780	0.004**	2.39	0.013*
chr_14_15,580,903	14	15,580,903	0.07	1.46	0.138
chr_14_15,613,280	14	15,613,280	0.06	1.44	0.144
chr_14_15,619,394	14	15,619,394	0.06	1.44	0.144

^*^Significant at α  =  0.05.

**Significant at α  =  0.01.

^a^Position of SNP in the *Cucurbita moschata* cv. Rifu.

## Discussion

While multiple sources of resistance to *Phytophthora* crown rot have been described in *Cucurbita*^9,10,12^, genomic regions (QTL) associated with this resistance are currently unknown. Markers tightly linked to such QTL would facilitate MAS for *Phytophthora* crown rot resistance, thus reducing phenotyping costs and accelerating genetic gain. In the current study, QTL-seq was successfully applied to identify three QTLs associated with *Phytophthora* crown rot resistance on chromosome 4 (*QtlPC-C04*), 11 (*QtlPC-C11*) and 14 (*QtlPC-C14*). The three loci were detected regardless of the parent used as a consensus reference genome, thus validating the reliability of QTL-seq as a rapid tool for QTL detection.

Although the population size (n = 168) was relatively small compared to those (n = 262–531) used in other crops for similar studies^15,17,18,20^, the bulk size employed (n = 20) was adequate to detect major loci involved in resistance. The coverage (45X) obtained in current study is within the range (6X− 80X) reported for other successful QTL discovery studies^15,17,18,20^.

The detection of three independent (non-interacting) QTLs (*QtlPC-C04*, *QtlPC-C11* and *QtlPC-C14*) in the current study supports previous findings by Padley *et al*.^12^ that three independent dominant genes (R1R2R3) are involved in *Phytophthora* crown rot resistance in *C. moschata*. Padley *et al*.^12^ concluded that the three genes must be present in homozygous or heterozygous state to confer resistance against the pathogen. However, data reported here suggests that the three genes are not always required to confer resistance against *Phytophthora* crown rot because the susceptible parent (Butterbush) contributed alleles for resistance at two of the loci (*QtlPC-C04* and *QtlPC-C14*) (Fig. 4). Instead, we propose that *QtlPC-C11* (resistance from #394-1-27-12) confers incomplete dominance for resistance, such that homozygous and heterozygous genotypes at this locus lead to resistant and intermediate resistance, respectively. Indeed, #394-1-27-12, which is homozygous at *QtlPC-C11* but lacks alleles for resistance from *QtlPC-C04* and *QtlPC-C14*, is highly resistant (Fig. 4). The proportion of individuals in the resistant bulk that were homozygous for the resistance allele at *QtlPC-C11* (marker chr_11_4,702,536) was 0.35, and this marker was significantly associated with resistance (P < 0.05). Majority of F_2_ individuals homozygous for the resistant allele at *QtlPC-C11* ranged from 0–2, suggesting that other loci not identified in the current study may contribute to *Phytophthora* crown rot resistance (Supplementary Table 2). Typically, with the QTL-seq method, loci explaining ≥ 10% of phenotypic variation can be detected using bulk sizes of 15% of the total F_2_ population^15^. In the current study, the bulks represented 11.9% of the total population size, which is in the range (3–11%) of previous quantitative trait mapping studies^15,17,18,20^; however, this may have been insufficient to detect QTL of minor effect for our specific trait. Branham *et al*.^21^ reported similar results in watermelon, where minor QTL for resistance to *Fusarium* wilt remained undetected when using a small bulk size (3% of the total population) in QTL-seq.

Individuals with a heterozygous genotype at *QtlPC-C11* could only confer resistance in presence of resistance alleles (either homozygous or heterozygous state) from *QtlPC-C04* and *QtlPC-C14*, which potentially act as modifiers for resistance. The F_1_ individuals, which are heterozygous (Fig. 4) at *QtlPC-C04*, *QtlPC-C11* and *QtlPC-C14*, were resistant to *Phytophthora* crown rot. Similarly, a high proportion of F_2_ individuals that were heterozygous at *QtlPC-C11* (chr_11_4,702,536), but homozygous or heterozygous for the resistant allele at *QtlPC-C04* (chr_04_2,050,610) and *QtlPC-C14* (chr_14_15,580,903), showed resistance to *Phytophthora* crown rot. This three-marker genotype combination was significantly associated with resistance in the F_2_ population (P < 0.0001).

To the best of our knowledge, this is the first report on QTL associated with *Phytophthora* crown resistance in *C. moschata*. The results presented here indicate that *QtlPC-C11* is a good candidate for MAS targeting *Phytophthora* crown rot resistance, and that markers linked to this QTL (chr_11_4,702,536, chr_11_4,811,256, chr_11_4,815,808, chr_11_4,825,468 and chr_11_5,102,780) may be utilized in the breeding program. However, since the QTLseq study was conducted based on phenotype data of single F_2_ individuals, these markers must be validated in independent populations to allow replicated screening. *QtlPC-C11* confers resistance to *Phytophthora* crown rot in an incomplete dominance mechanism; therefore, breeders may consider targeting *QtlPC-C04* and *QtlPC-C14* to augment resistance. Functional analysis of the resistant gene homologs identified within confidence intervals of the three QTLs will provide insight into the molecular mechanisms underlying resistance to *Phytophthora* crown rot in *C. moschata*.

## Methods

### Plant material and inoculum preparation

A cross was made in the greenhouse between breeding line #394-1-27-12 (resistant; paternal) and Butterbush (susceptible; maternal). A single F_1_ was selfed to generate an F_2_ population (n = 168). Inoculum for the experiment was prepared from a virulent isolate (#121) of *P. capsici* (provided by Dr. Pamela Roberts, University of Florida) grown on 14% V8 agar plates (140 ml V8 Juice, 3 g CaCO_3_, 16 g Agar per liter) agar petri dishes (100 × 15 mm) under constant fluorescent light at 28 °C for 10 days.

### Phenotyping

Seeds of parents and the F_1_ (n = 16, each), and those of the F_2_ (n = 168) were sown in 4-inch pots containing sterilized Proline C/B growing mix (Jolly Gardener, Quakertown PA) amended with 14N-4.2P-11.6 K controlled-release fertilizer (Osmocote; Scotts, Marysville, OH). At the second true leaf stage, the seedlings were inoculated by burying a 0.5 cM^2^ agar plug around the crown of each plant, followed by a second inoculation with another agar plug 7 days later. A 0–5 rating scale for disease severity modified from Padley *et al*.^9^ was used in which 0 = no symptoms, 1 = small brown lesion at base of stem, 2 = lesion has expanded 1–2 cm from the original point of infection, 3 = lesion has progressed up to the cotyledons causing constriction at the base and plant has partially collapsed with apparent wilting of leaves, 4 = plant has completely collapsed with severe wilting present, and 5 = plant dead. Final disease severity was recorded at 28 days post-inoculation.

### DNA extraction, library preparation and whole genome re-sequencing

DNA was extracted from emerging first true leaf of the parents, and twenty most (DS = 0) and twenty least (DS ≥ 4) resistant F_2_ progeny using the FavorPrep Plant DNA kit (Ping-Tung, Taiwan) according to the manufacturer’s instructions. DNA concentration was determined using NanoDrop 8000 (Thermo Fisher Scientific, Waltham, MA), and equal amounts (500 ng) from each of the 20 individuals constituting a bulk were pooled. Library (2 × 150 paired-end) construction and whole genome re-sequencing of the parents and the two bulks was performed on the Illumina HiSeq X (Illumina, Inc., San Diego, CA) at the BGI sequencing center (Shenzhen, Guangdong, China).

### QTL-seq analysis

Adapter trimming and removal of reads containing more than 50% low quality bases (quality value ≤ 12) was performed at BGI. The quality of Fastq sequences provided were further explored using FastQC tool (Babraham Institute, Cambridge, England). Sequence coverage was approximated using the formula C = LN/G, where C is coverage, G is the haploid genome length of squash (~372 Mb), L is the read length, and N is the number of reads that mapped to the reference genome. Best practices for variant calling were employed for mapping the sequences to a reference genome and calling variants using Genome Analysis Toolkit (GATK)^26^. Briefly, the raw reads where aligned to the *C. moschata* cv. Rifu reference genome^27^ using BWA-MEM^28^. SAMtools^29^ was used for checking the alignment, sorting, and indexing the BAM files. Grouping and duplicate read identification were performed with Picard Tools (http://broadinstitute.github.io/picard/). Finally, GATK was used to realign suspicious intervals, and to call and filter variants. Consensus fasta files (“consensus reference genomes”) for each of the parents (#394-1-27-12 and Butterbush) were built using SAMtools mpileup by replacing *C. moschata* cv. Rifu reference alleles with the respective parent allele across all loci^15,22^. The final vcf files were converted to*.table* format using *VariantsToTable* tool for analysis in R^30^.

The QTLseqr R package^31^ was used to detect QTL. The input SNP file was filtered based on average coverage per sample, such that each SNP had a read depth of no less than 50 for each bulk. The cutoff was determined by exploring the data with read depth histograms and following the recommended QTLseqr guidelines. Setting a read depth of 50 per bulk excluded 26,609 and 25,705 SNPs out of the 1,069,408 and 980,881 called SNPs after alignment to Butterbush and #394-1-27-12 consensus reference genomes, respectively. For each bulk, the SNP-index across all loci was calculated as the proportion of reads that were different from the parental reference allele^15^. The delta (Δ) SNP-index was calculated by subtracting the SNP-indices of the bulks at each loci. Calculations for SNP-indices were performed separately with each parent serving as the consensus reference genome. Identification of candidate QTL regions was performed using a 1 Mb sliding window in R^30^, whereby the confidence intervals for the ΔSNP-indices was determined using 10,000 simulations.

### Marker development and association with resistance

For each candidate QTL region, polymorphic indel and SNP markers with the highest ΔSNP-index in the QTL regions were targeted for primer design. Genetic sequences flanking target markers were extracted from the Cucurbita moschata cv. Rifu^27^ reference genome. Primers were designed using Primer3Plus^32^. In total, eleven markers were targeted, ten indels and one SNP as a dCAPS (Supplementary Table 1). The parents, F_1_ and individuals comprising the resistant (n = 20) and susceptible (n = 20) bulks were genotyped with all markers using gel electrophoresis.

The Kruskal-Wallis test (P = 0.05) was used to test the association of eleven markers with *Phytophthora* crown rot resistance in the susceptible and resistant bulks (Fig. 4), then eight of these markers were used to genotype the entire F_2_ population (n = 168, Supplementary Table 2). The association of the genetic markers with disease resistance was further explored with the R/qtl package^33^ following recommended procedures^34^. The *est.map* function was used to estimate a genetic map for the markers. Following data exploration with R/qtl, non-parametric interval mapping was selected for QTL mapping and implemented using the *scanone* function (model = “np”, method = “imp”), where each marker was tested independently to determine if there was a QTL at that position. The genome wide likelihood of the odds (LOD) scores were determined by running 4,000 permutations and the 99, 95, and 90 percentiles of the distribution were used as thresholds. The *scanone* function was used to calculate the LOD scores, and to determine marker significance. In order to test possible interactions between QTL, Multiple QTL mapping was performed. Interaction plots from the *scantwo* function in combination with models plotted with *makeqtl*, *fitqtl* and *stepwiseqtl* functions were used to determine significant QTL interaction.

### Candidate genes

For each significant QTL interval, candidate nucleotide-binding sites leucine-rich repeat (NBS-LRR), serine/threonine protein kinase (KIN) and protein phosphatase (PP) resistance gene-homologs were identified using the *Cucurbita moschata* cv. Rifu reference genome^35^.

## Supplementary information

Supplementary Figures

Supplementary Table 1.

Supplementary Table 2.
