# Supplementary material for: QTL-seq for identification of loci associated with resistance to Phytophthora crown rot in squash
Source: Sci Rep. 2020 Mar 24;10:5326. doi: 10.1038/s41598-020-62228-z (PMC7093484; doi:10.1038/s41598-020-62228-z)

# QTL-seq for identification of loci associated with resistance to *Phytophthora* crown rot in squash

Alexis Ramos, Yuqing Fu, Vincent Michael, and Geoffrey Meru

**Supplementary Figure 1** QTLseq outputs across 20 *Cucurbita moschata* chromosomes using Butterbush (susceptible) consensus reference genome for mapping. The red line represents the tricube∆SNP for the ∆SNP index. The green, purple, and blue lines are the 90%, 95%, and 99% confidence intervals for the regions, respectively.

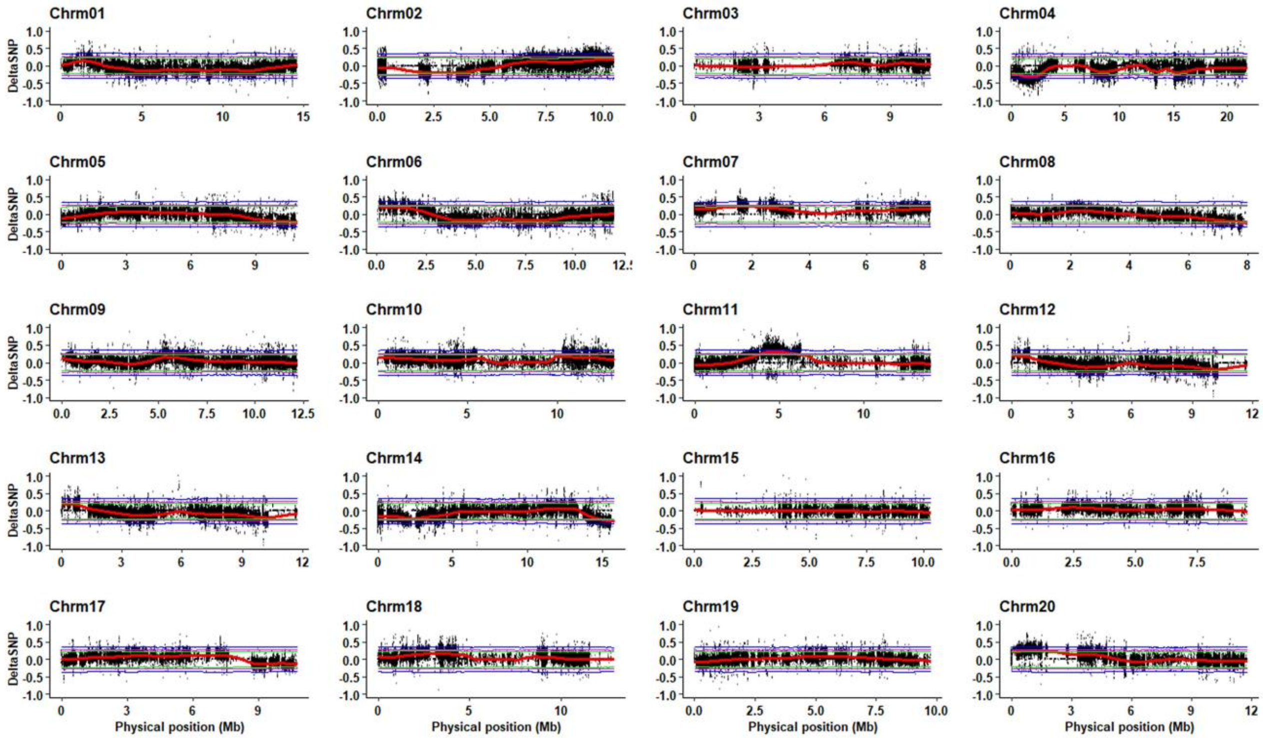

**Supplementary Figure 2** QTLseq outputs across 20 *Cucurbita moschata* chromosomes (Chrm) using #394-1-27-12 (resistant) consensus reference genome for mapping. The red line represents the tricubeΔSNP for the ΔSNP index. The green, purple, and blue lines are the 90%, 95%, and 99% confidence intervals for the regions, respectively.

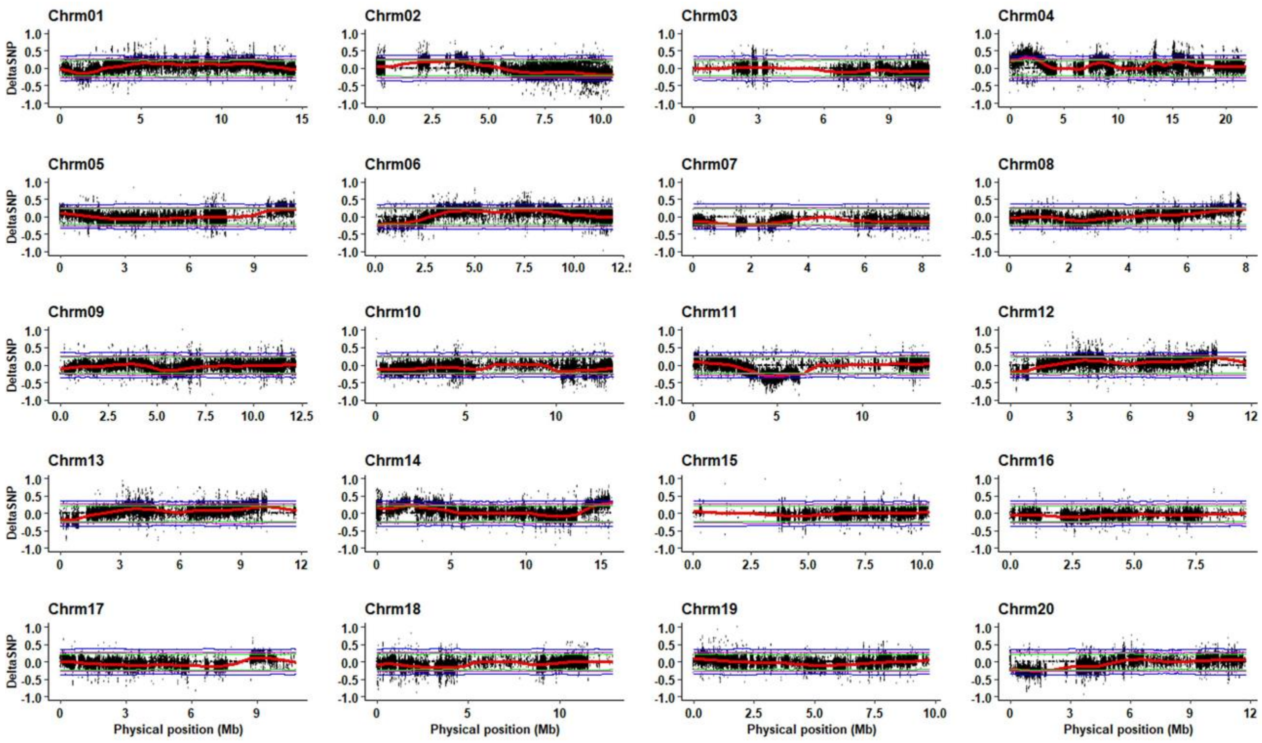

Supplement: Supplementary file 1 — Supplementary Figures [file 41598_2020_62228_MOESM1_ESM.pdf]
